# MiR-26a-5p as a Reference to Normalize MicroRNA qRT-PCR Levels in Plasma Exosomes of Pediatric Hematological Malignancies

**DOI:** 10.3390/cells10010101

**Published:** 2021-01-08

**Authors:** Carlotta C. Damanti, Enrico Gaffo, Federica Lovisa, Anna Garbin, Piero Di Battista, Ilaria Gallingani, Anna Tosato, Marta Pillon, Elisa Carraro, Maurizio Mascarin, Caterina Elia, Alessandra Biffi, Stefania Bortoluzzi, Lara Mussolin

**Affiliations:** 1 Maternal and Child Health Department, Padova University, 35128 Padova, Italy; carlottacaterina.damanti@studenti.unipd.it (C.C.D.); federica.lovisa@unipd.it (F.L.); anna.garbin.1@studenti.unipd.it (A.G.); pierodibattista.phd@gmail.com (P.D.B.); ilaria.gallingani@studenti.unipd.it (I.G.); anna.tosato@studenti.unipd.it (A.T.); alessandra.biffi@unipd.it (A.B.); lara.mussolin@unipd.it (L.M.); 2 Istituto di Ricerca Pediatrica Città della Speranza, 35127 Padova, Italy; 3 Department of Molecular Medicine, Padova University, 35121 Padova, Italy; enrico.gaffo@unipd.it; 4 Pediatric Hematology, Oncology and Stem Cell Transplant Division, Padova University Hospital, 35128 Padova, Italy; marta.pillon@unipd.it (M.P.); elisa.carraro87@gmail.com (E.C.); 5 Pediatric Radiotherapy Unit, Centro di Riferimento Oncologico (CRO) di Aviano, IRCCS, 33081 Aviano, Italy; mascarin@cro.it (M.M.); celia@cro.it (C.E.); 6 CRIBI Interdepartmental Research Center for Innovative Biotechnologies (CRIBI), Padova University, 35131 Padova, Italy

**Keywords:** circulating microRNAs, exosomes, qRT-PCR, normalization, reference genes, hematological malignancies, miRNA, lymphoma

## Abstract

Plasma exosomal microRNAs (miRNAs) are considered as valid circulating biomarkers for cancer diagnosis and prognosis. Quantitative real-time polymerase chain reaction (qRT-PCR), the most commonly used technique to assess circulating miRNA levels, requires a normalization step involving uniformly expressed endogenous miRNAs. However, there is still no consensus on reference miRNAs for plasma exosomal miRNA abundance normalization. In this study, we identified a panel of miRNAs with stable abundance by analyzing public plasma exosome RNA-seq data and selected miR-486-5p, miR-26a-5p, miR-423-5p and miR191-5p as candidate normalizers. Next, we tested the abundance variation of these miRNAs by qRT-PCR in plasma exosomes of healthy donors and pediatric patients with anaplastic large cell lymphoma, Burkitt lymphoma, Hodgkin lymphoma and mature B-cell acute lymphoblastic leukemia. MiR-486-5p and miR-26a-5p showed the most stable levels, both between healthy controls and patients and among the malignancies analyzed. In light of previous reports on miRNA stability in different exosome isolation methods, our data indicated that miR-26a-5p is a bona fide reference miRNA for qRT-PCR normalization to evaluate miRNA abundance from circulating plasma exosomes in studies of hematological malignancies.

## 1. Introduction

The use of liquid biopsy is of high interest in cancer research as a valuable noninvasive source of biomarkers [1]. Among different biological materials that circulate in the bloodstream, such as circulating tumor cells, cell-free DNA and RNA, proteins and metabolites, extracellular vesicles (EVs) are considered as the most promising carriers of circulating biomarkers. The term EVs covers a broad spectrum of cell-derived, membrane-enclosed particles that can originate from any type of body fluid, including urine, amniotic fluid, ascites, saliva and blood [2,3]. An important category of EVs is represented by exosomes, 40–150 nm endosome-derived vesicles originating from multivesicular bodies [4]. Exosomes are involved in cell–cell communications as they transfer proteins, lipids, DNAs, messenger RNAs (mRNAs) and noncoding RNAs originating from the source cell to different recipient cells [5,6]. Exosomes have recently attracted the interest of oncologists because the exosomal cargo is rich in disease biomarkers [7]. MicroRNAs (miRNAs) enriched in serum and plasma exosomes have been suggested as promising diagnostic and prognostic biomarkers for cancer, in view of their relatively high stability [8] and their well-known role as post-transcriptional regulators of gene expression [9]. In hematological malignancies [10], several studies have demonstrated the pathogenetic importance of exosomal miRNAs and suggested their use for diagnostic and prognostic purposes [11]. For example, Feng et al. found an increased expression of miR-99a-5p and miR-125-5p in plasma exosomes of diffuse large B-cell lymphoma associated with chemoresistance and poorer prognosis of patients [12].

Quantitative real-time polymerase chain reaction (qRT-PCR) is routinely used to detect circulating levels of miRNA [13]. To compare the miRNA expression variation between different conditions (such as health and disease), a normalization step is necessary, which requires the simultaneous measurement of a reference miRNA using the ΔΔCT method [14]. In general, so-called “housekeeping molecules” with stable expressions, such as the small nucleolar RNAs SNORD44 (RNU44), SNORD48 (RNU48) and the nuclear RNA RNU6-1 (U6) are extensively used as reference elements for miRNA quantification in cell and tissue samples. Instead, in consideration of their variable expressions in plasma and serum [15,16,17], they are not suitable for normalizing circulating miRNAs. In accordance with this, U6 levels were found highly variable in sera of healthy individuals, patients with critical illness and liver fibrosis [15], suggesting that disease conditions can affect the level of this small RNA. In principle, miRNAs with a stable expression in healthy and disease conditions can be used as normalizers. As known, the expression of most miRNAs is tissue-dependent [18,19,20] and miRNA expression is affected by disease and cancer. Moreover, there is no consensus on standard reference miRNAs for qRT-PCR normalization, especially for plasma exosomes. The introduction of an exogenous miRNA as spike-in control has been proposed as a possible normalization strategy [8]. However, this approach only allows the control of technical biases related to sample preparation without ensuring the adjustment for biological and other technical variability factors [17,21].

In this study, we evaluate the expression stability of different miRNAs in plasma exosomes derived from healthy donors and pediatric patients with different hematological malignancies and identify a reliable reference miRNA for this clinical context.

## 2. Materials and Methods

### 2.1. Public Small RNA-Seq Datasets and Bioinformatic Analysis

We retrieved small RNA-seq data of healthy donor (HD) plasma exosomes from GSE128359, which included data of two independent studies, and GSE100467 datasets deposited in the Gene Expression Omnibus (GEO) database [22]. In these studies, exosomes were isolated with the ExoQuick exosome precipitation solution (System Biosciences, Palo Alto, CA, USA) or the Total Exosome Isolation Reagent and Total Exosome RNA and Protein Isolation Kit (Life Technologies, Carlsbad, CA, USA). Samples with fewer than two million sequenced reads were discarded, and we randomly selected an equal number of samples from each dataset and obtained a total of 69 HD plasma exosome samples.

Small RNA-seq data were processed with miR&moRe2 v0.2.3 [23] to identify and quantify microRNAs. Small RNAs with read sums across samples of ≥ 10 and detected in ≥ 80% of the samples were further processed, as they were considered consistently expressed. Raw read counts were normalized with DESeq2 [24] and the limma v3.42.2 R/Bioconductor package [25] was used to correct for batch effects. The corrected normalized miRNA expression estimates were used to calculate a modified Z-score for each miRNA, as in Peltier et al. [26].

### 2.2. Plasma Samples Collection

Peripheral blood samples of pediatric lymphoma/leukemia patients were collected from patients enrolled in treatment protocols of the Associazione Italiana di Ematologia e Oncologia Pediatrica (AIEOP). Written informed consent was obtained from parents or legal guardians of each patient before enrolment. Peripheral blood samples with written informed consent were also collected from healthy donors and considered as the control group. The study was approved by the ethics committee of each participating institution. A total of 72 plasma samples were collected from pediatric patients at diagnosis of anaplastic large cell lymphoma (ALCL; *n* = 14), Burkitt lymphoma (BL; *n* = 15), Hodgkin lymphoma (HL; *n* = 15), mature B-cell acute lymphoblastic leukemia (mALL; *n* = 15) and HD (*n* = 13). Finally, we collected plasma from 23 ALCL pediatric patients before the last chemotherapy cycle of the ALCL99 treatment protocol [27] as a follow-up time point.

Plasma was obtained by blood centrifugation at 820× *g* for 10 min. The supernatants were carefully removed and centrifuged again at 2500× *g* for 10 min to minimize blood cell contamination.

### 2.3. Plasma Exosome Isolation

For each sample, two plasma fractions of 500 µL each were independently filtered with a 0.22 µm filter (Spin-X Centrifuge Tube Filter, Corning Incorporated, Corning, NY, USA) and then used for exosome isolation by a MISEV 2018 [28] approved protocol using the exoRNeasy Midi kit (Qiagen, Hilden, Germany) according to the manufacturer’s instructions, which allows the obtention of an exosome enriched fraction by membrane affinity columns. One of the exosome enriched fractions was used for exosome characterization, and the other for RNA extraction.

### 2.4. Exosome Characterization

The isolated exosomes (see Section 2.3) were eluted in 300 μL of XE buffer and quantified by using the FluoroCet Exosome Quantitation Kit (System Biosciences, Palo Alto, CA, USA). Exosomes were also assessed by Nanoparticle Tracking Analysis (NTA) and by transmission electron microscopy (TEM). NTA was conducted on a Nanosight NS300 instrument (Malvern Panalytical, Malvern, UK). The instrument was equipped with a 488 nm laser, a high sensitivity sCMOS camera and a syringe pump. The plasma exosome samples were mixed by vortexing and subsequently diluted to 1:1000 in particle-free PBS 1X to obtain a concentration within the recommended measurement range (1–10 × 10^8^ particles/mL). Experiment videos were analyzed using NTA 3.1 build 54 software (Malvern Panalytical) after capture in script SOP Standard Measurement (3 videos of 60 s per measurement), using a syringe pump speed of 30. A total of 1500 frames were examined per sample. TEM analysis with Tecnai G2 Spirit microscope (FEI Company, Hillsboro, USA) was performed on pellets of purified exosomes loaded on formvar/carbon-coated grids. Ammonium-Molybdate (2%) was used as a standard negative stain in biological electron microscopy before mounting in the sample position of the microscope. Exosomes were diluted to form a thin layer on the EM grid to afford the transmission of the electron beam. A Tecnai G2 Spirit TEM was used to image exosome samples with diameters between 30 and 150 nm and a magnification of up to 300 kX.

### 2.5. Exosomal RNA Extraction and qRT-PCR

The exosomes isolated (see Section 2.3) were eluted in 700 µL of Qiazol (Qiagen) for RNA extraction. RNA was extracted with the exoRNeasy Midi kit (Qiagen) following the manufacturer’s instructions. RNA quality was then assessed by an Agilent 2100 Bioanalyzer (Agilent Technologies, Santa Clara, CA, USA).

MiRNA retrotranscription was performed by using the TaqMan™ Advanced miRNA cDNA Synthesis Kit (Thermofisher Scientific, Waltham, MA, USA), following the manufacturer’s protocol. The amount of RNA isolated from plasma exosomes was at the lower bound of standard quantification methods’ sensitivity range. Thus, a fixed volume of 2 μL of RNA per reaction was used to obtain an equal loading of samples, as recommended by the TaqMan™ Advanced miRNA cDNA Synthesis Kit protocol. In the first step of retrotranscription, 10 pM of exogenous Caenorhabditis elegans cel-miR-39 was added as a technical control for the reaction efficiency. MiRNA expression levels were evaluated by qRT-PCR with TaqMan™ Advanced miRNA assays (hsa-miR-26a-5p ID_477995_mir, hsa-miR-486-5p ID_478128_mir, hsa-miR-423-5p ID_478090_mir, hsa-miR-191-5p ID_477952_mir, and cel-miR-39 ID_478293_mir; Thermofisher Scientific). The RT reaction products were used in 5 μL PCR reactions at a final dilution of 1:10. qRT-PCR reactions were run, with 3 replicates, on a ViiA™ 7 Real-Time PCR System using these cycling conditions: hold 20 sec, 95 °C; 95 °C, 1 sec and 60 °C, 20 sec for 40 cycles.

### 2.6. Statistical Analysis

Statistical analysis was performed using GraphPad Prism 7 (GraphPad Software, La Jolla, CA, USA, www.graphpad.com). A Shapiro–Wilk normality test was performed to evaluate the distribution of the samples. For statistical analysis comparing two groups, we used an unpaired t-test (for normally distributed data) or Mann–Whitney test (non-normally distributed data). To test more than two groups, one-way ANOVA on ranks (ordinary one-way ANOVA for normally distributed or Kruskal–Wallis test for non-normally distributed values) was used to compare Ct values among groups; Dunn’s test for nonparametric distributions and Tukey’s test for parametric distribution were used for pairwise comparisons. Statistical significance was set to *p*-value ≤ 0.05 in all tests.

## 3. Results

### 3.1. Plasma Exosomal miRNAs Defined by the Analysis of Public Small RNA-Seq Datasets

We searched the GEO database for small RNA-seq datasets of plasma exosomes to explore the exosomal small RNA cargo with a genomic approach. A set of 69 HD samples was compiled and further analyzed focusing on the miRNA fraction. Overall, 58 miRNAs were detected and inspected to identify those with high and stable levels across samples. The miRNAs were ranked by abundance variation according to a modified Z-score that also accounts for the relative abundance of miRNA (see Materials and Methods) and assigned higher scores to miRNAs with high abundance and low variability across the samples. We identified the 20 miRNAs top-ranked by Z-score (Table 1).

**Figure 1:**
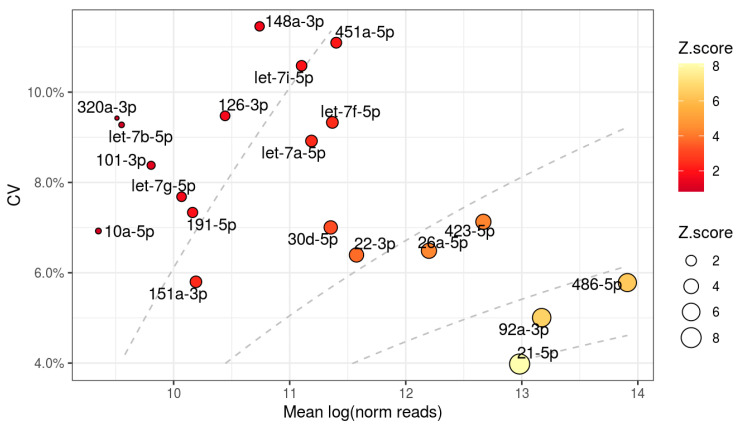
Relationship between abundance and variation of the 20 most stably expressed in plasma exosomes of healthy donors (HDs). Dashed lines represent two-unit intervals of Z-scores in the range 2 to 8 (top left to bottom right). CV: coefficient of variation.

MiR-21-5p, miR-92a-3p and miR-486-5p obtained the highest scores, as they were highly abundant and with low variation (Figure 1). Further in the ranking, miR-26a-5p and miR-423-5p showed sizable abundance and slight variation. It is worth noting that miR-423-5p was ranked higher than miR-22-3p despite having higher variation, showing the contribution of the miRNA abundance on the computation of the Z-scores and the following ranking.

### 3.2. Expression Levels of Candidate Reference miRNAs in Plasma Exosomes

Following the Z-score ranking obtained with the explorative RNA-seq data analysis, we considered the identified miRNAs for abundance stability assessment with qRT-PCR in plasma exosomes of pediatric lymphoma and leukemia patients and HDs.

MiR-21-5p and miR-92a-5p, top-ranked according to the RNA-seq analysis, were excluded from validation because of their well-known oncogenic role [29,30], which would make them potential targets for experimental validation in cancer studies instead of normalization factors. In particular, miR-21-5p is a diagnostic marker in tumor tissue of diffuse large B-cell lymphoma [31] and plays an oncogenic role in HL and BL [32,33]. Similarly, miR-92a-5p has been reported to play a role in lymphoproliferative disorders, especially in B-cell lymphomas. This miRNA belongs to the miR-17~92 cluster, whose expression is controlled by the MYC transcription factor and triggers the proliferation of tumor cells [34]. Therefore, we selected miR-486-5p, miR-26a-5p and miR-423-5p for the qRT-PCR validation analysis. We also included miR-191-5p because it has been described as a stable miRNA in sera and plasma of various neoplasias [35,36,37] and is recommended by two different biotechnology companies as one of the miRNA normalizers for plasma and serum samples [38,39].

The abundance of miR-486-5p, miR-26a-5p, miR-423-5p and miR-191-5p was investigated in plasma exosomes from HDs and in lymphoma/leukemia samples. Plasma exosomes characterized by Nanosight and TEM showed the typical exosome size distribution with a peak at 105 nm (Figure S1). The number of exosomes obtained from an equal starting plasma volume of different donors was not significantly variable (Figure S2), indicating that the use of equal volumes of all samples is appropriate for miRNA quantification. Electropherograms of exosomal RNAs showed that the samples were enriched in small RNAs (Figure S3). All samples showed good retrotranscription efficiency as measured by exogenous cel-miR-39 amplification, with Ct values ranging from 14 to 19 (Figure S4a). Average Ct values ranged from 22.26 of miR-26a-5p to 24.84 of miR-423-5p, confirming a good expression level for each miRNA analyzed (Figure S4b–e).

**Figure 2:**
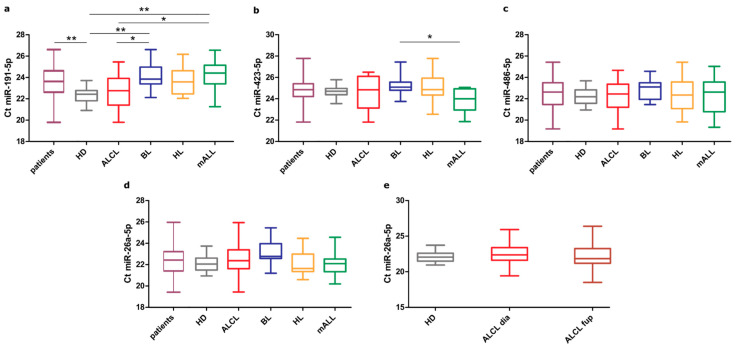
Expression (Quantitative real-time polymerase chain reaction (qRT-PCR) Ct values) of candidate reference miRNAs in plasma exosomes from healthy donors (HDs), representing control samples, and from pediatric patients with different hematological malignancies (ALCL: anaplastic large cell lymphoma; BL: Burkitt lymphoma; HL: Hodgkin lymphoma; mALL: mature B-cell acute lymphoblastic leukemia). (**a**–**d**) Ct of samples from HDs compared with patients considered altogether (patients) and separately, by disease. Ct distribution of normally distributed values (miR-486-5p, miR26a-5p and miR-191-5p) were compared by unpaired t-test or Tukey test, whereas miR-423-5 *p* values were compared by nonparametric Mann–Whitney or Dunn’s test. (**e**) Expression of miR-26a-5p in HD, ALCL at diagnosis (ALCL dia) and at follow up (ALCL fup). Only the statistically significant differences of Cts are marked. *: *p* < 0.05; **: *p* < 0.01.

To identify the most stable miRNA under the conditions considered, the differential expression among the groups was evaluated. In accordance with the RNA-seq data, we confirmed a low variability in HDs for the four analyzed miRNAs, while wider expression ranges were observed in the patients. When comparing the patients with HDs, we found a significantly different expression for miR-191-5p, which was higher in the patients (Figure 2a). Further, when each disease group and HD were considered separately, miR-423-5p and miR-191-5p showed significant variable expressions across the sample groups (Table S1). In particular, miR-423-5p was more abundant in BL than in mALL (Figure 2b), while miR-191-5p had a significantly different abundance in several sample group contrasts (Figure 2a). On the contrary, when comparing the miR-486-5p and miR-26-5p expression levels between the groups, no significant difference was detected (Figure 2c,d), suggesting that these two miRNAs are stably expressed in HDs and patients of the four different lymphoma/leukemia subtypes considered. Overall, these results suggest that miR-486-5p and miR-26-5p are suitable normalizers for miRNA quantification in plasma exosomes. Finally, we explored whether therapy can affect miR-26a-5p abundance in plasma exosomes. Measurements of miR-26a-5p in samples of ALCL patients collected before the last chemotherapy cycle showed that its abundance did not change compared to HDs and the level at diagnosis (Figure 2e), confirming that miR-26a-5p load in exosomes is highly stable.

## 4. Discussion

Vesicle-enclosed circulating miRNA studies using qRT-PCR aim to disclose noninvasive biomarkers depending on the choice of an endogenous reference miRNA to normalize the measured abundances. In the present work, we focused on a series of pediatric hematological malignancies—namely ALCL, BL, HL, and mALL—as representative of the most common histological subtypes of the pediatric age [40], and we investigated a panel of miRNAs suitable for plasma exosomal miRNA expression normalization.

Despite the high number of studies on circulating vesicle-enclosed miRNAs, small RNA-seq data of exosomes are still scarce in public datasets. Nevertheless, we found a sizable sample set that suited our explorative analysis from two pioneering independent studies, which used two different exosome isolation commercial kits. These data provided a genome-wide picture of the sRNA exosomal cargo in healthy individuals, which allowed us to establish a group of miRNAs with high and stable abundance among several healthy donors. Our miRNA ranking score took into account both expression variability and expression level since a sufficiently high abundance is as crucial as stability for a candidate normalizer miRNA in qRT-PCR assays.

From a panel of four miRNAs with stable expressions in HD without an obvious involvement in known tumorigenic processes, we identified miR-486-5p and miR-26a-5p as the most stable miRNAs both between HD and patients and among hematological disease subtypes. MiR-26a-5p has previously been shown to be stable in serum exosomes of carcinoma patients [18] and supernatants of cardiosphere-derived cells and adipose tissue-derived mesenchymal stem cells [19,41]. It is worth noting that in these studies both ultracentrifugation and commercially available kits for exosome extraction (Norgen Urine Exosomes Isolation kit -NorgenBiotek Corp.-, miRNeasy Mini Kit -Qiagen-) were used, suggesting that miR-26a-5p levels are independent of the extraction method (ultracentrifugation vs. commercial kits) and the exosome source (serum or cell supernatants). Conversely, it has been shown that miR-486-5p expression is influenced by the exosome isolation method, showing lower levels when using ultracentrifugation than ExoQuick isolation (System Biosciences) [42].

Additionally, we investigated miR-191-5p as it is indicated as a stable plasma miRNA in other studies and by manufacturing companies [35,36,37]. Moreover, miR-191-5p was listed among the top 20 most stable miRNAs resulting from our analysis of public RNA-seq data. Notably, we found that miR-191-5p expression was significantly different in our samples, suggesting that miR-191-5p, reportedly stable in total plasma and serum, is variable in plasma exosomes instead. This result corroborates our initial hypothesis that specific calibrator miRNAs are needed for exosomal miRNA normalization and that the choice of the commonly used plasma reference miRNAs can bias the analysis and lead to wrong conclusions.

Overall, our data indicated that both miR-486-5p and miR-26a-5p are suitable reference miRNAs for expression normalization across samples of plasma exosomes from patients with hematological malignancies and HD. However, miR-26a-5p is preferable because it is stable regardless of the origin of the exosome sample and the isolation method, whereas miR-486-5p quantification can be significantly influenced by the exosome isolation method, thus limiting its use in meta-analyses or comprehensive comparative studies. Lastly, chemotherapy did not affect miR-26a-5p levels in plasma exosomes, indicating its suitability as a reference for miRNA kinetics studies during therapy.

In this study, four representative types of pediatric lymphomas were analyzed to obtain a comprehensive picture of this cancer. Additionally, the inclusion of one subgroup of pediatric leukemia expanded the spectrum of hematological diseases considered. Further analysis will be needed to assess the suitability of miR-26a-5p as a normalizer for qRT-PCR assays of plasma exosomes in other leukemia subtypes as well as in other tumors, to understand whether miR-26a-5p could be applied also in nonhematological contexts.

## Figures and Tables

**Table 1:** Top 20 microRNAs (miRNAs) most stably expressed in plasma exosomes of 69 healthy donor samples according to the Z-score of expression measures. CV: coefficient of variation.

MicroRNA	Z-Score	CV	Rank
miR-21-5p	8.14	3.98	1
miR-92a-3p	6.66	5.01	2
miR-486-5p	6.38	5.78	3
miR-26a-5p	4.32	6.49	4
miR-423-5p	4.31	7.13	5
miR-22-3p	3.78	6.40	6
miR-30d-5p	3.24	7.00	7
let-7f-5p	2.44	9.33	8
let-7a-5p	2.42	8.92	9
miR-151a-3p	2.39	5.80	10
miR-451a-5p	2.07	11.10	11
let-7i-5p	1.98	10.58	12
miR-191-5p	1.86	7.34	13
miR-126-3p	1.68	9.48	14
let-7g-5p	1.67	7.68	15
miR-148a-3p	1.60	11.46	16
miR-101-3p	1.25	8.38	17
miR-10a-5p	0.89	6.93	18
let-7b-5p	0.87	9.28	19
miR-320a-3p	0.82	9.43	20

## Data Availability

All data are included in the paper.

## Supplementary Materials

The following are available online at https://www.mdpi.com/2073-4409/10/1/101/s1, Figure S1: exosome size distribution; Figure S2: the number of exosomes in plasma samples; Figure S3: electropherograms of exosomal RNAs; Figure S4: Descriptive statistics of Ct values; Table S1: Statistical analysis summary.
