# Supplementary material for: MiR-26a-5p as a Reference to Normalize MicroRNA qRT-PCR Levels in Plasma Exosomes of Pediatric Hematological Malignancies
Source: Cells. 2021 Jan 8;10(1):101. doi: 10.3390/cells10010101 (PMC7827902; doi:10.3390/cells10010101)
Supplement: Supplementary file 1 [file cells-10-00101-s001.pdf]

MiR-26a-5p as a reference to normalize microRNA qRT-PCR levels in plasma exosomes of pediatric hematological malignancies

Carlotta C. Damanti [1,2,†], Enrico Gaffo [3,†], Federica Lovisa [1,2], Anna Garbin [1,2], Piero Di Battista [1,2], Ilaria Gallingani [1,2], Anna Tosato [1,2], Marta Pillon [4], Elisa Carraro [4], Maurizio Mascarin [5], Caterina Elia [5], Alessandra Biffi [1,2], Stefania Bortoluzzi [3,6,§,*] and Lara Mussolin [1,2,§]

[1] Maternal and Child Health Department, Padova University, Padova, Italy
[2] Istituto di Ricerca Pediatrica Città della Speranza, Padova, Italy
[3] Department of Molecular Medicine,Padova University, Padova, Italy
[4] Pediatric Hematology, Oncology and Stem Cell Transplant Division, Padova University Hospital, Padova, Italy
[5] Pediatric Radiotherapy Unit, Centro di Riferimento Oncologico (CRO) di Aviano, IRCCS, Aviano, Italy
[6] CRIBI Interdepartmental Research Center for Innovative Biotechnologies (CRIBI), Padova University, Padova, Italy
[†] These authors have contributed equally
[§] Co-last authors
* Correspondence: stefania.bortoluzzi@unipd.it; Tel.: +39-049-827-6502

SUPPLEMENTARY MATERIAL

Figure S1. (a) Transmission electron microscopy (TEM) of vesicles obtained from an ALCL plasma sample; **(b)** the particle diameter (nm) distribution of vesicles isolated from an ALCL plasma sample, as measured by Nanoparticle Tracking Analysis (Nanosight, Malvern Panalytical, Malvern, UK).

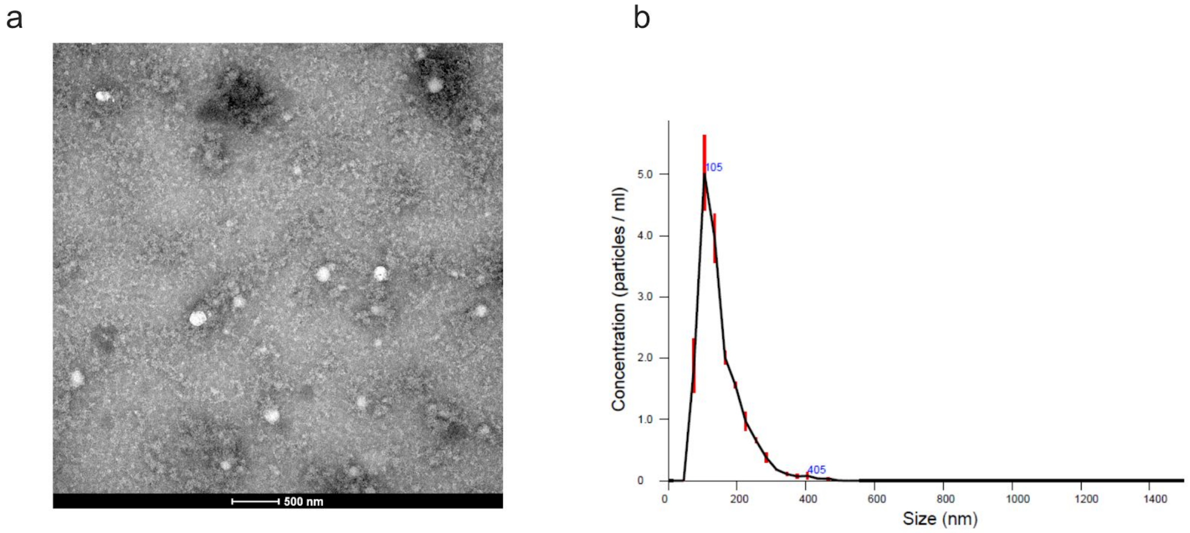

Figure S2. The number of exosomes in 500 µl of plasma samples of healthy donors (HD; n=4), pediatric anaplastic large cell lymphoma (ALCL; n=14) and Hodgkin lymphoma (HL; n=6). No statistically significant difference was detected (p>0.05) neither in multiple comparisons among HD, ALCL and HL (Tukey's multiple comparison test) nor between HD and the patients altogether (unpaired t-test).

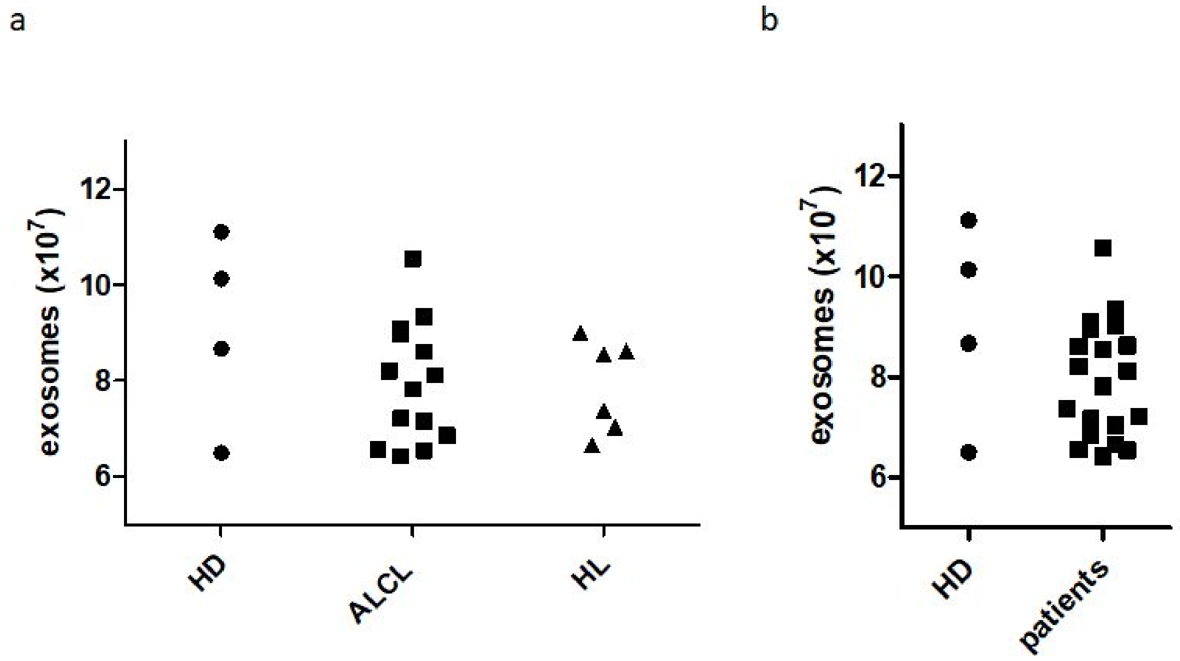

Figure S3. (a) Plasma exosomal RNA assessment of quality by Bioanalyzer pico-chip in 15 samples. HD: healthy donors; ALCL: anaplastic large cell lymphoma; BL: Burkitt lymphoma; HL: Hodgkin lymphoma; mALL: mature B cell lymphoblastic leukemia. **(b)** Plasma exosomal RNA detection of miRNAs by Bioanalyzer small RNA chip on three representative samples.

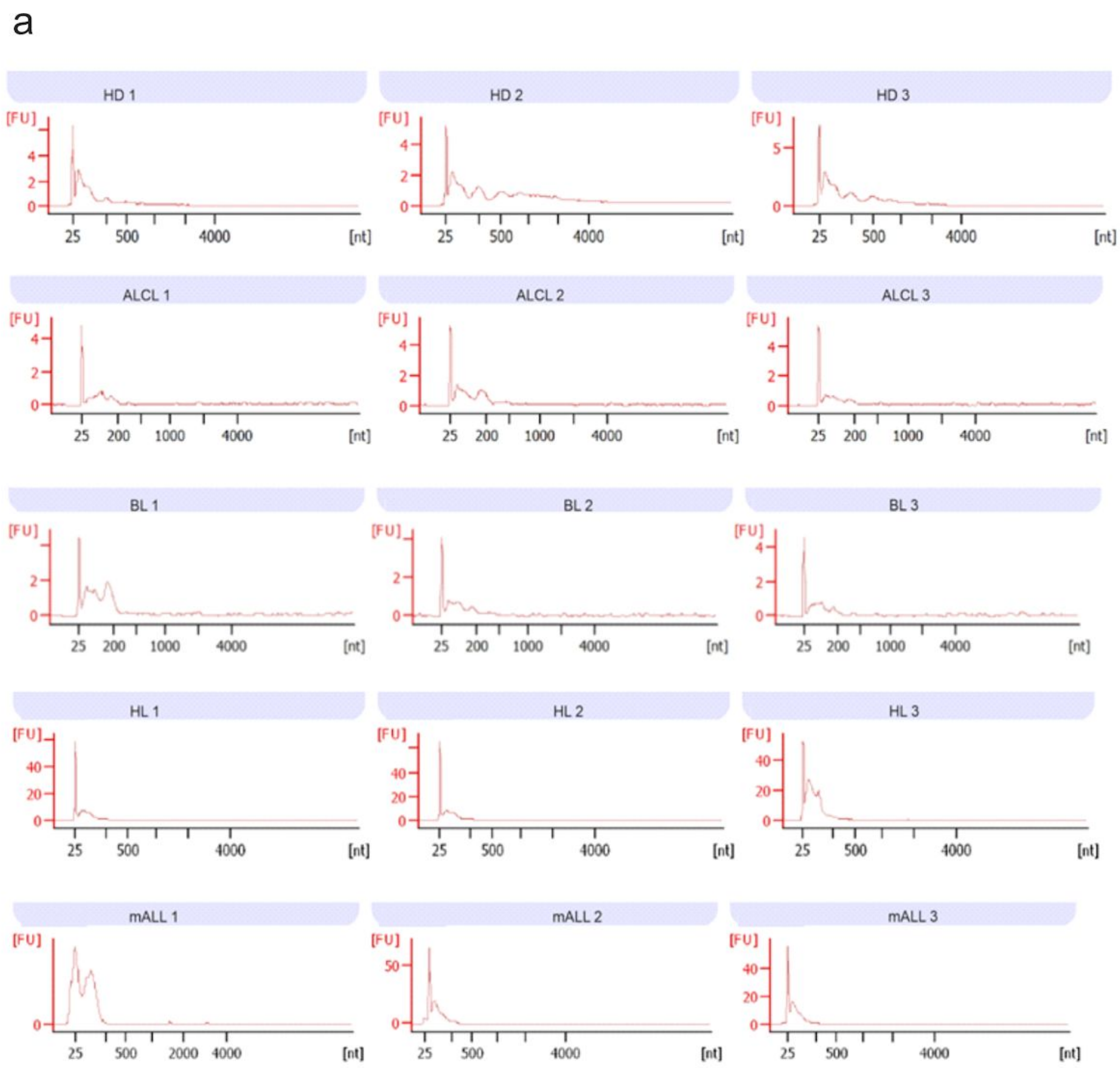

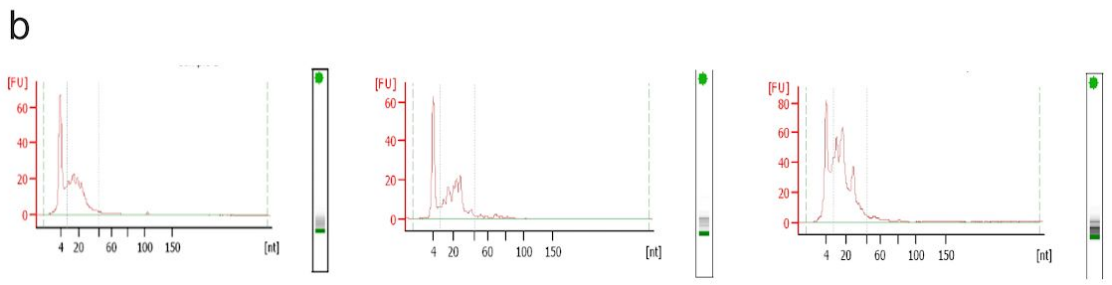

Name	From [nt]	To [nt]	Average Size [nt]	Size distribution in CV [%]	Conc. [pg/µl]	% of Total	Co lor
Small RNA	0	322	14	86.5	1,700.4	100	
miRNA	10	40	19	34.6	1,604.3	94	

Name	From [nt]	To [nt]	Average Size [nt]	Size distribution in CV [%]	Conc. [pg/µl]	% of Total	Co lor
Small RNA	0	311	18	89.8	2,042.7	100	
miRNA	10	40	22	31.0	1,928.2	94	

Name	From [nt]	To [nt]	Average Size [nt]	Size distribution in CV [%]	Conc. [pg/µl]	% of Total	Co lor
Small RNA	0	310	29	100.0	3,071.4	100	
miRNA	10	40	19	37.5	2,780.7	91	

Figure S4. Descriptive statistics of Ct values obtained for each candidate miRNA and cel-miR-39, representing all the analysed samples.

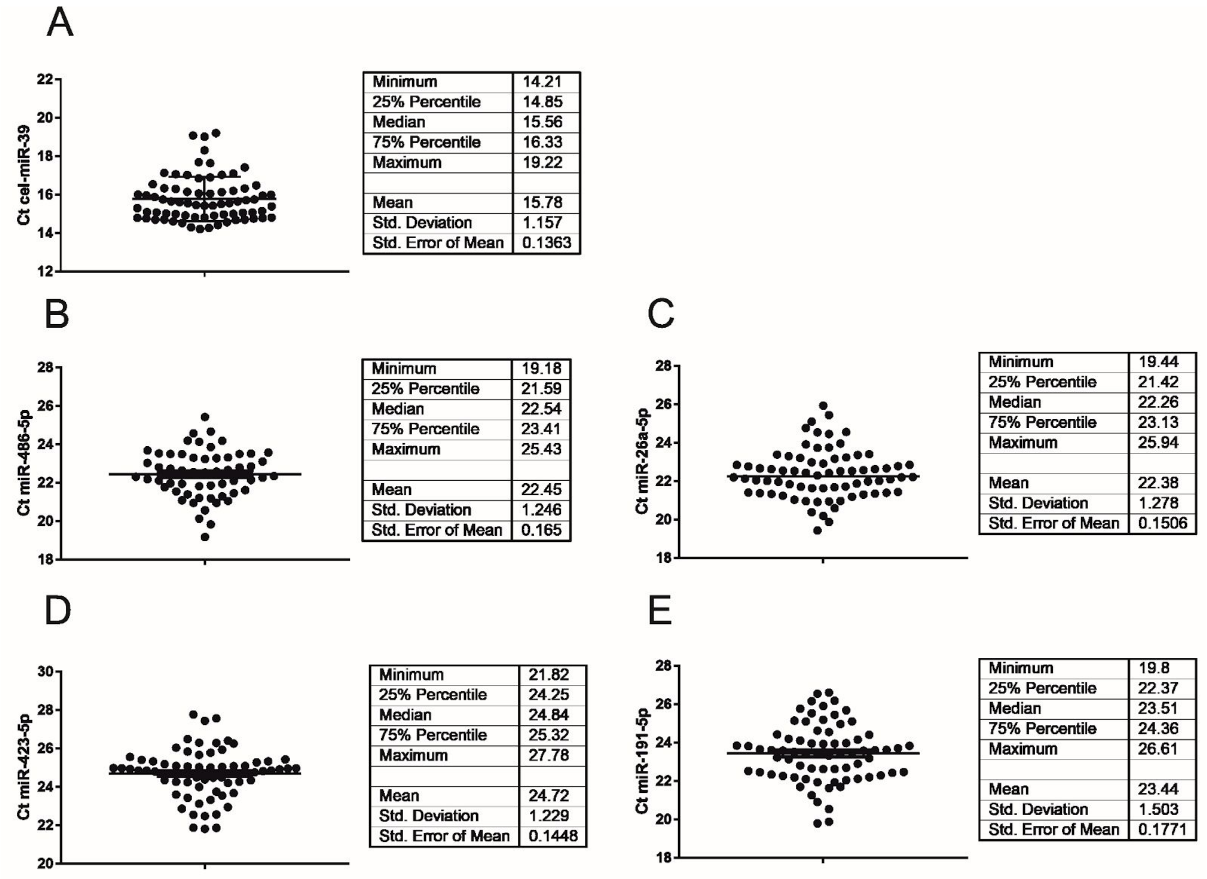

Table S1. Statistical analysis summary.

	miR-486-5p		miR-26a-5p		miR-423-5p		miR-191-5p	
**Normality test**	*P value*	*Normal distribution*	*P value*	*Normal distribution*	*P value*	*Normal distribution*	*P value*	*Normal distribution*
	0,9953	yes	0,3756	yes	0,0258	no	0,5556	yes
**one-way ANOVA**	*P value*	*Summary*	*P value*	*Summary*	*P value*	*Summary*	*P value*	*Summary*
	0,7456	ns	0,1178	ns	0,0244	*	0,0009	***
**ANOVA multiple comparisons**	*Adjusted P Value*	*Summary*	*Adjusted P Value*	*Summary*	*Adjusted P Value*	*Summary*	*Adjusted P Value*	*Summary*
HD vs. ALCL	>0,9999	ns	0,9392	ns	>0,9999	ns	0,9746	ns
HD vs. BL	0,748	ns	0,2595	ns	>0,9999	ns	0,0087	**
HD vs. HL	0,9998	ns	>0,9999	ns	>0,9999	ns	0,0814	ns
HD vs. mALL	0,9967	ns	0,9992	ns	>0,9999	ns	0,007	**
ALCL vs. BL	0,8133	ns	0,7021	ns	>0,9999	ns	0,0408	*
ALCL vs. HL	>0,9999	ns	0,8984	ns	>0,9999	ns	0,2624	ns
ALCL vs. mALL	0,9995	ns	0,8357	ns	0,6515	ns	0,0336	*
BL vs. HL	0,8208	ns	0,1867	ns	>0,9999	ns	0,9079	ns
BL vs. mALL	0,9008	ns	0,1378	ns	0,0174	*	>0,9999	ns
HL vs. mALL	0,9997	ns	0,9999	ns	0,1307	ns	0,8793	ns

	**miR-26a-5p**	
**Normality test**	*P value*	*Normal distribution*
	0,0672	yes
**one-way ANOVA**	*P value*	*Summary*
	0,7603	ns
**ANOVA multiple comparisons**	*Adjusted P Value*	*Summary*
HD vs. ALCL dia	0,8127	ns
HD vs. ALCL fup	0,9999	ns
ALCL dia vs. ALCL fup	0,7735	ns
